# Unique double guidewire technique using a guidewire perforating the diverticulum for a difficult biliary cannulation

**DOI:** 10.1055/a-2086-2153

**Published:** 2023-05-26

**Authors:** Kazuya Koizumi, Kento Shionoya, Sakue Masuda, Jun Kubota, Karen Kimura

**Affiliations:** Gastroenterology Medicine Center, Shonan Kamakura General Hospital, Kamakura, Kanagawa, Japan

Biliary cannulation of an intradiverticular papilla, classified type 1 as per the Li-Tanaka classification, is sometimes challenging 1 . Although in cases with difficult biliary cannulation the pancreatic duct guidewire placement technique is useful 2 3 , placing the guidewire in the pancreatic duct is sometimes difficult. We report a successful case of biliary cannulation in a patient with an intradiverticular papilla using an unusual double guidewire technique taking advantage of a guidewire perforating the diverticulum.

An 80-year-old woman presented to another hospital with high fever and abdominal pain. She was diagnosed with cholangitis due to common bile duct stones ( Fig. 1 ). Three attempts of endoscopic retrograde cholangiopancreatography (ERCP) failed to identify the papilla. Therefore, she was referred to our hospital. Identification of the papilla was difficult; the orifice of the papilla was identified after cleaning food remnants in the diverticulum and inserting the tip of the side-viewing endoscope (TJF-Q290V; Olympus Medical Systems, Tokyo, Japan) into the diverticulum ( Fig. 2 ). Contrast medium injection was possible, and the bile duct stones and pancreatic duct were visualized. However, guidewire insertion into the bile and pancreatic ducts was challenging. During several attempts of wire-guided cannulation, the guidewire perforated the diverticulum through the papilla ( Fig. 3 ). We attempted to perform biliary cannulation using the double guidewire technique with the guidewire perforating the diverticulum instead of placing the guidewire in the pancreatic duct to help stabilize and visualize the orifice of the papilla. Wire-guided cannulation of the bile duct was successful ( Fig. 4 ), and the stone was removed using a basket and balloon catheter following endoscopic balloon dilation ( Video 1 ).

**Fig. 1:**
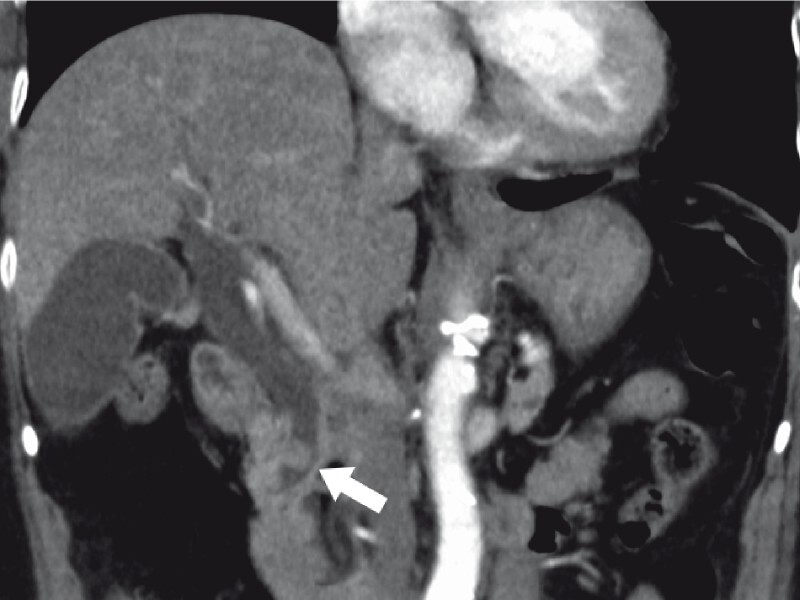
Computed tomography showing a common bile duct stone.

**Fig. 2:**
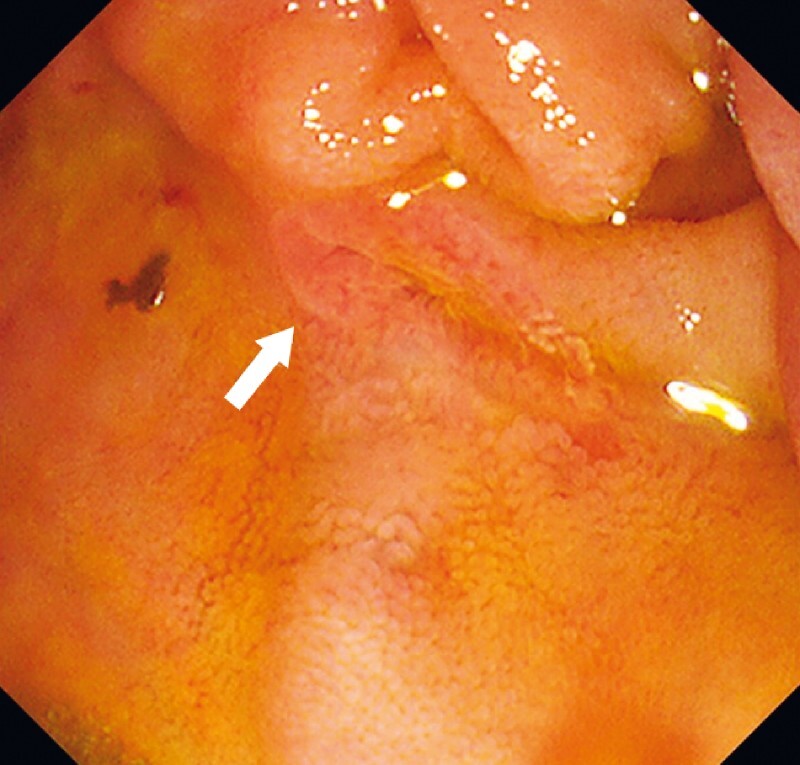
Endoscopic image: The orifice of the papilla could be identified after cleaning food remnants in the diverticulum and insertion of the tip of the side-viewing endoscope into the diverticulum.

**Fig. 3:**
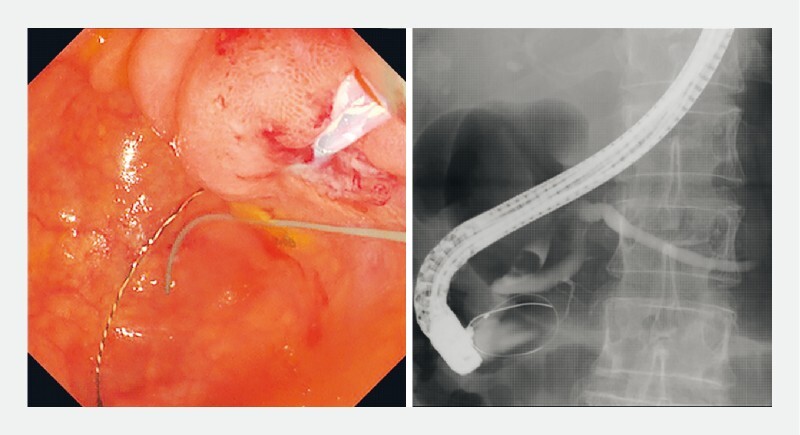
The guidewire perforating through the papilla into the diverticulum during several attempts of wire-guided cannulation.

**Fig. 4:**
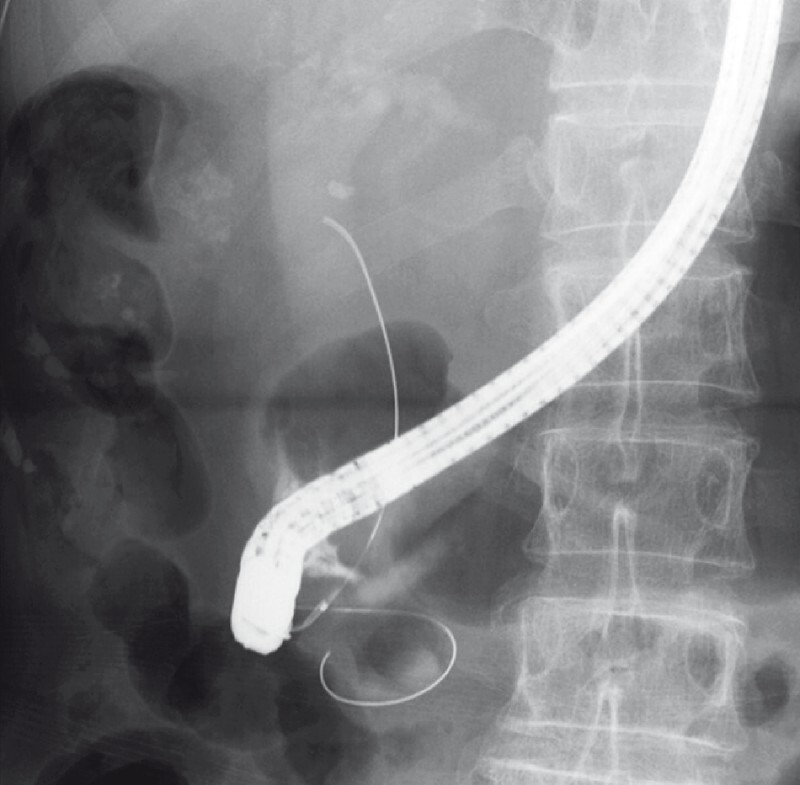
Fluoroscopic image: Using the double guidewire technique with the guidewire perforating the diverticulum, the wire-guided cannulation of the bile duct was successful.

**Video 1** Biliary cannulation was successfully performed using a unique double guidewire technique with a guidewire that had perforated the diverticulum.


The guidewire sometimes perforates the papilla during ERCP; however, most cases improve conservatively 4 5 . Therefore, instead of removing the guidewire that perforated the diverticulum, we used it for the double guidewire method to cannulate the bile duct.

Endoscopy_UCTN_Code_TTT_1AR_2AH

## Notes

Endoscopy E-Videos

https://eref.thieme.de/e-videos
*E-Videos* is an open access online section of the journal *Endoscopy* , reporting on interesting cases and new techniques in gastroenterological endoscopy. All papers include a high-quality video and are published with a Creative Commons CC-BY license. Endoscopy E-Videos qualify for HINARI discounts and waivers and eligibility is automatically checked during the submission process. We grant 100% waivers to articles whose corresponding authors are based in Group A countries and 50% waivers to those who are based in Group B countries as classified by Research4Life (see: https://www.research4life.org/access/eligibility/ ). This section has its own submission website at https://mc.manuscriptcentral.com/e-videos
